# “Push” versus “Pull” for mobilizing pain evidence into practice across different health professions: A protocol for a randomized trial

**DOI:** 10.1186/1748-5908-7-115

**Published:** 2012-11-24

**Authors:** Joy C MacDermid, Mary Law, Norman Buckley, Robert Brian Haynes

**Affiliations:** 1 School of Rehabilitation Science, McMaster University, Hamilton, Ontario, Canada; 2 Hand and Upper Limb Centre Clinical Research Laboratory, St. Joseph’s Health Centre, London, Ontario, Canada; 3 Department of Anaesthesia, McMaster University, Hamilton, Ontario, Canada; 4 Department of Clinical Epidemiology and Biostatistics and Department of Medicine, McMaster University, Hamilton, Ontario, Canada

**Keywords:** Knowledge translation, Evidence-based healthcare, Implementation science, Health informatics, Pain, Physician, Rehabilitation, Nursing, Psychology

## Abstract

**Background:**

Optimizing pain care requires ready access and use of best evidence within and across different disciplines and settings. The purpose of this randomized trial is to determine whether a technology-based “push” of new, high-quality pain research to physicians, nurses, and rehabilitation and psychology professionals results in better knowledge and clinical decision making around pain, when offered in addition to traditional “pull” evidence technology. A secondary objective is to identify disciplinary variations in response to evidence and differences in the patterns of accessing research evidence.

**Methods:**

Physicians, nurses, occupational/physical therapists, and psychologists (n = 670) will be randomly allocated in a crossover design to receive a pain evidence resource in one of two different ways. Evidence is extracted from medical, nursing, psychology, and rehabilitation journals; appraised for quality/relevance; and sent out (PUSHed) to clinicians by email alerts or available for searches of the accumulated database (PULL). Participants are allocated to either PULL or PUSH + PULL in a randomized crossover design. The PULL intervention has a similar interface but does not send alerts; clinicians can only go to the site and enter search terms to retrieve evidence from the cumulative and continuously updated online database. Upon entry to the trial, there is three months of access to PULL, then random allocation. After six months, crossover takes place. The study ends with a final three months of access to PUSH + PULL. The primary outcomes are uptake and application of evidence. Uptake will be determined by embedded tracking of what research is accessed during use of the intervention. A random subset of 30 participants/ discipline will undergo chart-stimulated recall to assess the nature and depth of evidence utilization in actual case management at baseline and 9 months. A different random subset of 30 participants/ discipline will be tested for their skills in accessing evidence using a standardized simulation test (final 3 months). Secondary outcomes include usage and self-reported evidence-based practice attitudes and behaviors measured at baseline, 3, 9, 15 and 18 months.

**Discussion:**

The trial will inform our understanding of information preferences and behaviors across disciplines/practice settings. If this intervention is effective, sustained support will be sought from professional/health system initiatives with an interest in optimizing pain management.

**Trial registration:**

Registered as NCT01348802 on clinicaltrials.gov.

## Background

Acute and chronic pain affects a large number of Canadians. The prevalence of chronic pain in Canadian adults has been estimated to range from 18–29% [1-4], with 60% experiencing major losses of occupation or function [5]. The 2005 Canadian Community Health Survey found that chronic pain affected 27% of community seniors [2]. In Canada, current healthcare spending to address this problem is $6 billion/year [5] and, with an aging population, is expected to rise to $10 billion/year by 2025.

There is considerable variation in practice and widespread patient dissatisfaction with the way pain is managed across the spectrum, from relatively benign conditions to terminal illness. For example, in low back pain [6], unresolved pain is a common reason for repeat visits. Inadequately managed pain in serious illness is the most common reason for requesting assisted suicide [7]. Conversely, there are societal concerns about the use of narcotic medications for pain management and the potential societal impacts of misuse [8].

Many clinicians are not aware of recent pain research, and misconceptions are common [9,10]. In fact, health professionals may begin clinical practice with insufficient knowledge about pain, as illustrated by an entry-level curriculum for physicians, nurses, and rehab professionals. The majority (68%) of programs did not specify designated hours for pain education (only 32.5% of the respondents could identify specific hours allotted for pain course content), and the total amount of time allocated to pain varied from 13 to 41 hours. Of interest, veterinarians spent twice as much time studying pain [11]. Studies that cross different types of pain and health professions indicate a large gap between evidence and practice—this is reflected by a high prevalence of low competence/confidence in pain management [12-19].

Pain management is central to health disciplines. Physicians, nurses, and rehabilitation professionals are commonly involved in managing acute and chronic pain [20]. Pain is one of the most common reasons for patients to consult these professions [21]. While scope of practice and focus may vary across professions, pain management provides an important context to study professional differences in use of evidence since pain is a high priority to all and an area where interprofessional practice is supported by evidence [22-26].

Evidence-based practice (EBP) can narrow the gap between research knowledge and practice [27]. Studies suggest that an EBP approach is used in nursing [28], medical [29], and rehabilitation practice [30-32] with variable success. Increased use of evidence-based approaches [33] or supports such as guidelines or decision aids is linked to improved clinical decision making and patient care [34,35], but the effects are not consistent [36,37] and, thus, cannot be assumed to work in all contexts.

Professional associations and individual clinicians in rehabilitation are highly supportive of EBP [30,32,38-45]. Despite positive attitudes and motivations about EBP, many studies have reported barriers to effective implementation of evidence from research in clinical practice [46]. Several studies indicate that clinicians experience time as an obstacle to searching for research evidence [47,48]. Clinicians also admit that they lack the skills required to navigate literature databases and to appraise medical literature effectively [32,49,50]. A lack of time [40-42] combined with inadequate searching and appraisal skills [20,21,43-45] are the primary barriers reported. It has been determined that it requires 53 minutes on average—divided between database searches (39 minutes) and obtaining the articles (25 minutes)—to answer a clinical question using a traditional EBP approach [51].

Traditional approaches to knowledge translation (KT) in EBP have focused on providing knowledge and technical EBP “skills” to practitioners, leaving them with the burden of searching and appraising evidence [40,52-56]. Studies suggest these interventions change knowledge about EBP, but not behavior [57,58]. Practitioners have limited success “pulling out” relevant quality evidence because they lack the required searching, filtering, and appraisal skills [40-43,51,54,59,60]. Even with training, the time demands are substantial [51,56]. Current tools provide primitive alerting services that do not target specific practitioners, but send information by “dumping” procedures. As a result, practitioners are overwhelmed with a large volume of information, most of which does not pertain to their practice area. Thus, either these services are not used or alerts are ignored. A basic flaw in both the training or dumping-out KT approaches is that they fail to resolve the essential barriers of time pressures and lack of appraisal/filtering skills.

Evidence-based decision making originated within medicine and evolved to other professions. Limited studies, mostly in the United Kingdom, have addressed differences in attitude or awareness across professions [61,62]. Familiarity with evidence resources is less than might be expected. In 2009, only 44% of urologists were unaware of the PubMed search engine, and only 14% used it regularly [63].

Systematic reviews suggest technology-based EBP can enhance care [34,64,65], although evidence is sporadic. Working directly with the Health Information Research Unit (HIRU) at McMaster University, we developed an approach to push out targeted, clinically relevant, high-quality research evidence to practitioners. This Premium LiteratUre Service (PLUS) [66,67] removes the burden of appraising research evidence, as technical experts perform basic critical appraisal, and then filters the high-quality studies and systematic reviews with ratings of relevance and interest from expert clinicians from the field to send only what is clinically relevant and new to the individual clinician. We know this approach increases uptake in physicians [66] and is valued by nurses [28] and rehabilitation users (pilot data). In a cluster randomized trial funded by the Canadian Institutes of Health Research, with physicians in Northern Ontario, PLUS was shown to increase the use of medical literature by 57% and substantially increase the depth of use of evidence-based resources [57]. One of the limitations in KT research on this topic to date has been the use of surrogate outcome measures of knowledge utilization. It can be challenging to measure how new knowledge is integrated into clinical decision making, particularly in areas where complex reasoning is required. This study is designed to address some of those deficiencies.

The primary objective of this randomized crossover trial is to evaluate the incremental effect of a PUSH evidence resource (Pain PLUS) across professions (physicians, nursing, rehabilitation, psychologists) as compared to a standard (PULL) approach. The primary outcomes are uptake and application of evidence, and they will be assessed at baseline, 3, 9, 15 and 18 months.

## Methods/design

**Figure 1:**
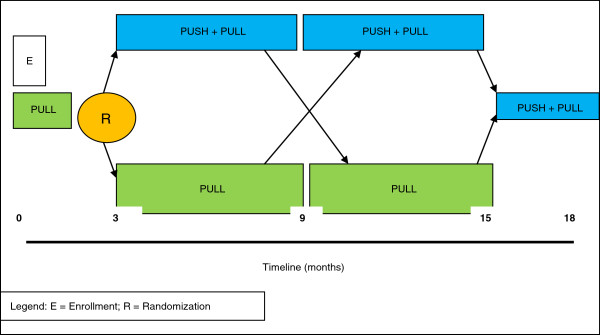
**Study Design** .

The trial is a crossover randomized controlled trial (RCT) with repeated baseline and follow-up measurements (Figure 1). The interventions are two different types of access to pain research evidence for four different types of professionals involved in pain management. The trial will begin with a three-month (repeated) baseline, during which average participant use of the standard PULL resource will be monitored. Participants will then be randomly allocated to either receive PUSH + PULL or continue to use the PULL resource. After six months, participants will cross over to the alternate intervention for an additional six months. To complete the trial, both groups will finish with three months of PUSH + PULL access. The repeated baseline period provides more stable estimates of participants’ access prior to randomization. The rationale for the crossover design is that it will maximize our statistical efficiency, increasing the precision of effect estimates. This is particularly important for our subgroup (profession) estimates; it will also provide better control of unknown potential confounders. The main potential drawback of a crossover design occurs if the “wash-out” is incomplete. While we will have control of the intervention delivery, the exposure to email alerts may affect use of the pull resources; therefore, we will test for order effects. The study received Ethics Approval from the McMaster University Research Ethics Board.

Traditional RCT methodology supports a single primary outcome measure, but a theoretical framework for outcome evaluation was based on the conceptual framework of technology-based KT [68,69]. Therefore, indicators of intervention effectiveness for this study are as follows:

1. Use of technology (structural evaluation): Embedded tracking will record the retrieval behavior (frequency of access; number of pain studies retrieved).

2. Perceived usefulness (subjective evaluation): Users will respond to randomly embedded judgments about the usefulness of their session experience.

3. Skills/behaviors in accessing information: An observational test of information-searching behaviors measuring how different professions access evidence for specific clinical questions and the type, quality, and usefulness of evidence retrieved.

4. Application: Evidence-based decision making

a. Participants’ EBP behaviors and decision making will be measured by a standardized self-report scale.

b. Chart-stimulated recall will measure competency for using an evidence-based approach to address problems within participants’ own clinical practice.

Our secondary objective is to evaluate professional differences in access/use of research. Therefore, we will evaluate whether the primary outcome measures—either at baseline or in response to the intervention—are modified by professional group. Further, we will perform a structured classification of the types of studies accessed by different professions to look at variations in the type of evidence valued.

We expect that (1) retrieval and application of pain evidence and satisfaction with evidence resources will improve more with push-out intervention and (2) there will be differences between professions in attitudes towards EBP, response to evidence tools/resources, and types of evidence most accessed.

### Interventions

**Figure 2:**
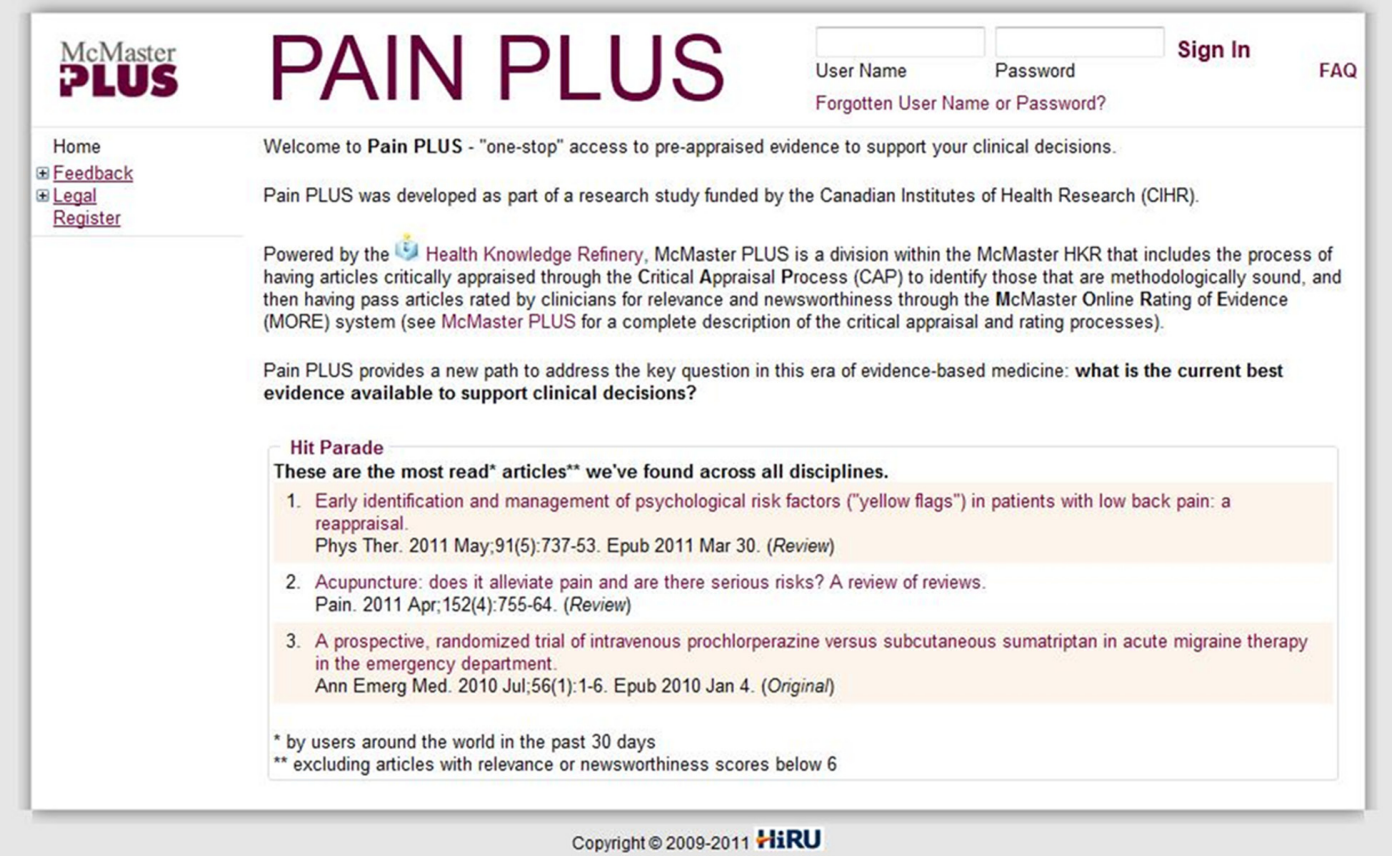
**Pain PLUS Intervention.** Screenshot of the Pain PLUS main webpage.

All participants will receive unique IDs and passwords and will be able to log into an evidence resource, Pain PLUS (Figure 2), to search for recent studies and systematic reviews of pain management (PULL). Once signed up, all practitioners will receive more specific instructions on using the evidence repository. This evidence repository has a cumulative collection of research evidence that has evolved over time—first for medicine, then for nursing, and then rehabilitation and psychology. It contains a searchable database with links to PubMed abstracts and direct links to any free full-text articles.

### PUSH + PULL intervention

**Figure 3:**
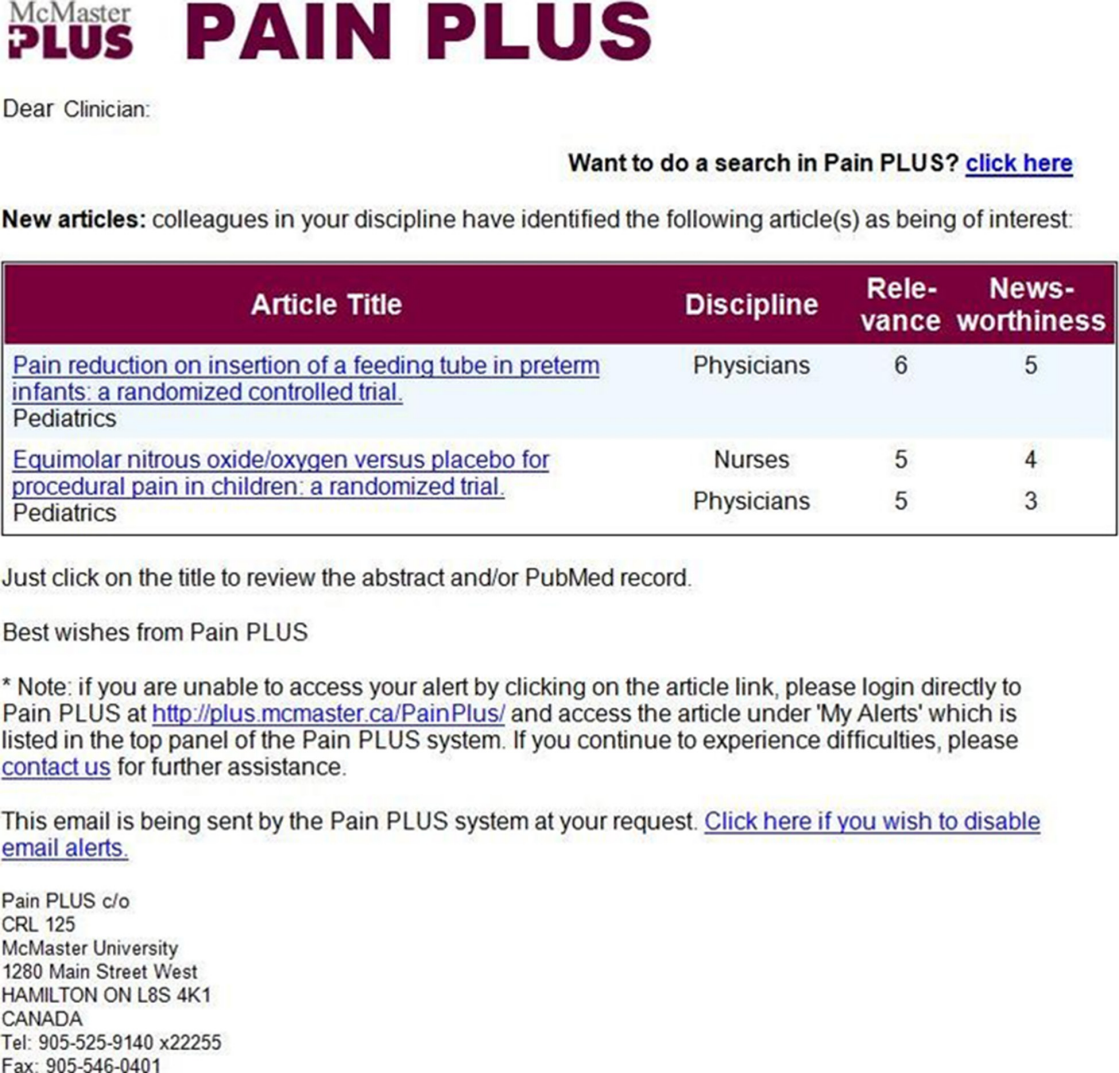
**Pain PLUS Intervention.** Screenshot of an example Pain PLUS email alert.

Pain PLUS is a customized, personalized, evidence-based, alerting and look-up service that links clinicians to new research findings most relevant to their clinical practice. This service is based on ongoing hand searches of over 120 clinical journals. Trained, experienced research staff determine scientific merit/quality rating. Discipline-specific clinical ratings of relevance and newsworthiness are obtained from volunteer raters who agree to screen abstracts. Raters are in active clinical practice and are recruited from the intervention target group communities (“educational influential”). All study participants will be signed into the *pain* content of the service but can also select email frequency and the cut-off for relevance and quality to control the degree of relevance and frequency of evidence they receive. Their experience will be a customized notification of new research (Figure 3).

### PULL intervention

Users will “see” a similar format when they access the pull intervention, but this “placebo control” website will not include any individualized information, nor will they receive alert emails. This group can access new research but must search for it.

### Randomization

Computerized randomization will allocate individuals to intervention group at three months; crossover is automatic at nine months.

### Study population and recruitment

Eligible practitioners must (1) be physicians, nurses, occupational therapists (OTs), physical therapists (PTs), or psychologists who are currently working in clinical practice at least one day/week; (2) be fluent in English; (3) have access to a computer at home or at work that has unrestricted access to the World Wide Web; and (4) have an active email account. Clinicians who meet the eligibility criteria are enrolled in the study by the research assistant.

### Outcome measures

We will use an evaluation framework developed specifically for technology-enabled knowledge translation [68-70] to evaluate our primary research question on KT effectiveness as outlined below and in Table 1. It is important when evaluating technology to determine whether the technology performs as expected (structural) and in a way that users find useful (subjective) to understand impacts on higher-level outcomes (cognitive and behavioral).

**Table 1:** Overview of Study Assessments

**Elements of evaluation based on a theoretical framework**[68-70]	**What is evaluated**	**Evaluation methods (applied throughout the trial)**
Structural	Technological functionality and design	1. Embedded quick questions on functionality
		2. Number of articles accessed.
Subjective (Usefulness)	Perceived usefulness of intervention	1. Embedded questions on usefulness of randomly selected logins.
Cognitive	1. Attitudes towards the use of evidence in practice	1. Attitudes about EBP from Knowledge/*Attitude*/Behaviour Questionnaire [71]
	2. Types of evidence valued	2. We will code using a taxonomy that addresses type of pain, type of intervention and type of research to look at preferences for information across disciplines.
Behavioral	Behaviour in Applying Evidence in Practice	
	1. Self-reported EBP behaviour	1. Self-reported from Knowledge/ Attitude/*Behaviour* Questionnaire [71]
	2. Research Access Skills	2. Skills at retrieving useful evidence when presented with a clinical question will be assessed by a structured online performance-based test of information access behavior [72]
	3. Application of Research Evidence in Clinical Decision-Making	3. Actual integration of use of evidence in decision-making will be assessed by chart stimulated recall [73-75]

### Structural

Structural usability analysis will have some embedded questions on functionality but will primarily be based on tracking of how the technology is used. We will have data on the number of logins and number of pain and other (specialty) research studies in the database that are accessed. These data will be summarized by the average frequency of use per month and mean number of studies accessed. Other utilization outcomes that will be tracked by the PLUS software include the change in the average frequency of use, average number of keystrokes per month, and the average number of times practitioners use certain specific electronic resources. Collection of these outcomes will be software regulated and, thus, not require any participant burden.

### Subjective

The subjective elements will focus on perceived usefulness of individual sessions. Participants will be asked at the end of each use to answer a single question with a 7-point scale, indicating the extent to which the service provided useful information. If some users fail to log off the system and invoke the final question, we will send them a query by email.

### Cognitive

#### Attitudes

There are few well-validated outcomes assessments of EBP, knowledge acquisition, or attitude/behavior change. In this study, cognitive elements will be assessed using a previously validated scale containing cognitive and behavioral questions specifically related to EBP [71]. This questionnaire has validated factor structure and construct validity and demonstrated responsiveness to EBP interventions [71]. We have added supplemental questions tailored specifically to Pain PLUS, adapted from the Evidence Updates trial [66] follow-up evaluations, for comparison purposes.

#### Behavioral

Behavior change is the ultimate goal of KT. It will be assessed through self-report, performance tests, and observer-based competency assessments. EBP behaviors will be self-reported using the Behavior Subscale of the Knowledge Attitudes and Behaviour Questionnaire (KABQ) validated in different professions [71]. Similar questions have demonstrated reliability and validity in nurses [76].

#### Skill at accessing research evidence

We will directly measure respondents’ skill in accessing information by providing a clinical scenario where a clinical question needs to be addressed and asking participants to search for research information to address that request. We will monitor their approach to searching and their satisfaction and intention to act upon the information acquired. This test will replicate previous methods used to compare information retrieval by physicians on Clinical Queries versus PubMed [72]. A subset of volunteers (30/profession) will search for information to address two posed clinical questions: one scenario will be a uniform multidisciplinary question (provided to all participants) and the other will be profession specific. Using our tracking software within Pain PLUS, we will be able to identify what search terms are used, what studies are located, the efficiency of their search strategies (*e.g.*, percent of relevant articles, time to find), and effectiveness of their search (quality of the relevant articles located). Volunteers will rate the usefulness of the research located. Our previous study determined that Clinical Queries was more efficient for physicians [72]. In this substudy, we will randomly allocate half of the participants to access through PubMed traditional search versus Clinical Queries, so we can assess whether Clinical Queries is more useful across professions. This subanalysis will provide more detailed and direct data on professional differences in accessing information.

#### Application of research evidence

We will obtain scores and a more detailed description of actual application of pain research evidence use before and after the intervention by performing chart-stimulated recall (CSR). This approach yields a rich and deep understanding of how evidence is used in practice based on semistructured interviews that probe how/why clinicians make choices about cases retrieved from their own records. A predetermined set of competencies are probed during the interview, and the performance is rated on a 7-point Likert scale. The score provides a quantitative estimate of competency (to use evidence in management of pain in their individual practice). We have successfully coded previous CSR interviews using content-analysis techniques. We have successfully used CSR in a KT study [73] to evaluate competency in clinician decision making, and others have indicated CSR is reliable and valid to assess different types of practice competency in physicians, nurses, and rehabilitation practitioners [73-75,77,78].

#### Timing of measures

The KABQ will be administered at baseline, 3, 9, 15, and 18 months. Other measures are embedded and distributed over time. CSR will be performed in a subset of 30/group during the first randomization cycle (at baseline and nine months). The skill assessment will be conducted in the final three months of the trial to allow maximum skill development to occur so that our comparisons of professional differences will be less affected by learning curves.

#### Mediators of use of evidence/intervention response

It is important to monitor and potentially adjust for important covariates that influence study outcomes. We will measure the following variables for each clinician: baseline knowledge/attitudes towards EBP, years of practice, highest degree, and comfort with technology. Organizational variables will also be collected at baseline, including rural/urban location, clinic size, access to online journals, peer support, administrative support, and management support. These variables will be entered into an analysis of covariance examining pre- and post-intervention effects. Our sample size is sufficiently large that we will be able to assess the primary effects of these variables, as well as interactions between organizational variables and participant variables.

#### Sample-size calculations

To determine the appropriate sample size for this trial, the following factors were considered: (1) the primary and secondary outcomes in the trial, (2) clinically significant differences for the outcomes based on our previous RCT, (3) Type I (alpha) and Type II (beta) errors, (4) estimates of variation from our pilot data, (5) study design, and (6) dropouts. These calculations represent sample size for the overall comparisons between the four professional groups and two intervention groups. Sample-size estimation was based on detecting an effect size of 0.40 between groups on any of the three aspects of outcome (usability, usefulness, and knowledge/attitude/behavior). Assuming Type I error = 0.05 (two-tailed), Type II error = 0.80, and the effect size = 0.40, the sample size required per group is 80. The sample size required for two comparison groups across four professions = 80 × 2 × 4 = 640. To account for our dropout rate (8/431), we have conservatively added 30 to recruit a sample of 670 practitioners. For analysis of response mediators, an *R*^2^ of 30% corresponds to an effect size of 0.42; 600 practitioners provides more than 99% power to detect this effect size with 12 predictors [79].

This study is organized locally but recruits participants internationally to inform practitioners of the study and the potential for free access to evidence resources. Our multimodal recruitment strategy will use professional associations, websites/conferences, and social media to inform practitioners of the study and the potential for free access to evidence resources.

### Proposed methods for protecting against other sources of bias

#### Control over eligibility violations

Participants will complete a baseline screen with the study research assistant, which will be independently reviewed by a trainee for violations. These will be reported (as a protocol diligence indicator), but no participants will be removed. No further rechecks of eligibility or exclusions will be included after this screening.

#### Control of biases through blinding

We have attempted to create a blinded control by creating a similar front-face to the website and blinding participants to the nature of the difference in the two resources (push versus pull), although we acknowledge differences will become apparent to users. Since usage data are embedded and automated, they are not influenced by data collectors or social bias. Research assistants and data analysts will be blinded to group allocation for the duration of the study and data analysis. After crossover, subjects will be aware of the difference and, therefore, no longer be blinded. Thus, the overall potential for bias due to lack of blinding has been minimized.

#### Control of contamination and co-intervention

Inside the study, participant crossovers are impossible since we control access to the intervention. Contamination that happens by participants accessing open access content for physicians by signing up under an alias for service is possible, but not probable. As part of enrollment, we will ask participants to refrain from using any new evidence access tools/services or EBP training during the study trial and will re-evaluate their reports of services used at the end of each intervention cycle. Co-intervention will be discouraged, including attendance at intensive EBP training (although we expect that routine exposure in settings is neither controllable nor a concern since these pre-existing efforts have not been effective).

#### Ensuring reproducibility and reliability of measurements of outcome

We will use reliable and valid measures of attitudes to EBP that have been used with the studied clinical disciplines [44,76,80,81]. CSR will be performed by two independent raters and checked for initial reliability.

#### Control of biases relating to (loss to) follow-up

We will use the following measures to ensure minimal loss to follow-up: (1) enrollment/consent will emphasize committing to the entire trial period and will exclude clinicians who cannot commit to the study for 18 months, (2) contact information will be updated at each study interval, and (3) response burden has been kept low. Burden is reduced since brief measures are embedded in the intervention; the total time required for extra questionnaires is relatively low and will be time distributed. The system will initiate regular reminders until the data collection is complete (or the participant withdraws consent).

#### Compliance

Compliance is one of our monitored variables. When participants fail to use an intervention, they are classified in some studies as noncompliant. We view lack of use as a failure of the intervention to meet participant needs. Thus, participants who do not use this service will not be classified as noncompliant or dropouts. Dropouts will be defined as participants who inform us that they no longer wish to participate in the study follow-up process. These participants will not be denied access to the intervention, and we will request permission to continue to use data from the embedded tracking of usage. Each participant will receive a $45 gift card upon completion of at least three survey assessments: baseline, one follow-up (at 3, 9, or 15 months), final assessment. Participants who complete the CSR or the search skills assessment will receive an additional $20 gift card as honorarium for time volunteered.

#### Planned data analyses

Quality checking of primary and secondary outcome data will be performed by re-entry of 100 cases and checking concordance. Descriptive statistics, including frequencies, means, and standard deviations, will be calculated and data displayed graphically for all study variables as a means of exploring data distributions. The data for all outcome measures will be analyzed to ensure that they meet the statistical assumptions necessary to use parametric statistics. Transformations may be considered if necessary to meet test assumptions. To test our main research question, we will use general, linear mixed-effects models [82] for repeated measurements over time to explore the relationship among the use/behavior scores, comparing the effects between professional groups and intervention types. The models will be fitted for the outcome scores over time as a function of the between-participant (profession/intervention) and within-participant (time) variables. Utilizing a mixed-model approach allows us to account for the dependence between outcome measurements taken over time from the same participant. The structure of the variance-covariance matrix, which dictates the dependence between measurements in the fitting of the model, will be chosen based on standard criteria in statistics. *Post hoc* pairwise comparisons of the two treatment arms at different time points will be done using multiple comparisons with adjustment to the Type I error rate. Type I error will be set to 5% (*i.e.*, α = 0.05) for all the calculation of the confidence intervals and performing the various hypothesis-testing procedures. Type I error will be adjusted accordingly for multiple comparisons. Standard diagnostic tools will be used to assess any of the model fittings. An intent-to-treat analysis will be employed—those who do not participate will be included in the analysis in their original group. No interim analyses are planned since there are no safety issues and we wish to ensure full power for both primary and secondary analyses.

We also plan to analyze difference in professional attitudes, evidence access, and use. We have powered our study to allow this analysis. Our subgroup analyses will also describe differences in types of evidence accessed. Our group previously determined that physicians prefer systematic reviews to primacy studies and that access was better through peer-reviewed journals than through Cochrane Collaboration [83]; we will now extend this type of analysis to look at evidence accessed across professions by comparing the types of primary studies (clinical, basic science; quantitative/qualitative), content (prognosis, diagnosis, clinical measurement, treatment effectiveness/outcomes, economic, etiology), and journals (disciplinary/interdisciplinary/other discipline).

### Steering committee

The Steering Committee will consist of the four investigators. The study coordinator will prepare a monthly report, detailing the accrual rate, loss to follow-up, withdrawals, and compliance with study protocols. Since this trial does not directly involve patients, there is no need for a Data Safety Monitoring Committee.

### Trial status

We have enrolled more than 70% of the sample target. More than 60% of the participants have reached the nine-month milestone (crossover).

## Discussion

This trial should provide unique information on evidence-access skills and professional differences in use of evidence. We expect to demonstrate the effectiveness of push-out targeted evidence (Pain PLUS) and to move the intervention into open access upon trial completion. We expect that some differences in evidence access and application will occur across professional groups, both in terms of types of pain interventions and types of research that are accessed.

It is our intent to ensure this intervention becomes open access to have ongoing impact. We believe this trial will inform our understanding on how to best deliver evidence to clinicians across a variety of professions dealing with pain. Sustainability of KT interventions is a critical issue area. As our findings emerge, we will deal with sustainability issues. Since evidence updates have been created for specific disciplines, including physicians, nurses, and rehabilitation professionals, it may be that folding in a pain resource into these existing resources would be the optimal method to ensure sustainability. Conversely, pain stakeholders may prefer a customized resource. A variety of pain resources are being developed, and strategies to share these across stakeholders are just emerging in stakeholder discussions. This may form an opportunity to tailor the knowledge from this study to emerging resources. Using the knowledge about what pain evidence exists, is accessed, and valued across professions will allow us to develop a pain knowledge strategy. The sustainability plan will be designed to maximize uptake of pain knowledge in a sustainable manner.

One of the benefits of this study is the ability to compare the evidence accessed by different professions when dealing with the same issue. We will use a descriptive approach to convey these differences by classifying the type of pain, the type of clinical questions, and the type of research design of these studies to convey if different types of evidence are valued by different professions. We anticipate, for example, that nurses and therapists might access more qualitative research, whereas physicians might access more RCTs on drug interventions. Psychologists are being included for the first time in this type of intervention, and their information behaviors are relatively unstudied. We expect that this information might help us understand if evidence scanning and filtering needs to be adjusted across professions. For example, decisions about how to filter qualitative studies may be more challenging than are such decisions in quantitative research.

We also acknowledge that the information resources that are available to knowledge users are rapidly expanding. Resources vary in their delivery, quality, customization, source content, and format of information. Knowledge users may become overwhelmed with information resources or stick with ones they have become comfortable with, irrespective of the quality of the information. It may not be clear to end users that the evidence-filtering process used in Pain PLUS protects them against low-quality information. Further, as clinicians often value advice about clinical decision making in actual cases, or implementation issues, they may be drawn to information resources that focus on these rather than the primary or synthesized research available. Since information is always in competition with other information, we cannot be certain that Pain PLUS will win in this competition.

## Competing interests

JCM, ML, and RBH were involved in the development of the Pain PLUS service. NB has no competing interests to declare.

## Authors’ contributions

JCM drafted the protocol. All authors participated in the study design and provided feedback on the protocol. All authors read and approved the final manuscript.
